# Weight Perception, Weight Control Intentions, and Dietary Intakes among Adolescents Ages 10–15 Years in the United States

**DOI:** 10.3390/ijerph16060990

**Published:** 2019-03-19

**Authors:** Andrea L. Deierlein, Alomi Malkan, Jaqueline Litvak, Niyati Parekh

**Affiliations:** 1 College of Global Public Health, New York University, New York, NY 10003, USA; am8140@nyu.edu (A.M.); jl7598@nyu.edu (J.L.); np31@nyu.edu (N.P.); 2 Department of Population Health, School of Medicine, New York University, New York, NY 10016, USA

**Keywords:** adolescent, weight, perception, diet, weight management, behaviors

## Abstract

Background: To examine associations of adolescents’ weight status perception and weight control intentions with dietary intakes. Methods: Cross-sectional data from adolescents aged 10–15 in the National Health and Nutrition Examination Surveys, 2005–2014 (n = 4940). Adolescents responded to questions regarding weight perception and if they were trying to change their weight. Intakes of calories, protein, carbohydrate, fat, saturated fat, sugar, and fiber were assessed using 24-h dietary recalls. Multivariable linear regression estimated associations of intakes with weight perception and weight control intentions. Results: The majority of adolescents perceived their weight as “about right”; however, 45% and 46% of boys and girls, respectively, reported trying to change their weight. Weight perception was not associated with intakes, with the exception of lower sugar (−13.65 g, 95% CI: −23.06, −4.23) and higher percent calories from protein (1.01%, 95% CI: 0.16, 1.87) in boys with overweight/obesity who perceived themselves as overweight, as well as lower percent calories from saturated fat (−1.04%, 95% CI: −2.24, −0.17) among girls with normal weight who perceived themselves as overweight. Weight control intentions were associated with intakes in boys only. Compared to boys who never tried to lose weight, boys who tried to lose weight consumed fewer calories (−188.34 kcal, 95% CI: −357.67, −19.01), a lower percent of calories from fat (−1.41%, 95% CI: −2.80, −0.02), and a greater percent of calories from protein (1.48%, 95% CI: 0.41, 2.55). Conclusions: Despite perceiving weight as “about right”, many adolescents reported trying to change their weight, which was associated with some dietary intakes. Efforts may be necessary to educate adolescents on healthy nutrition and weight management behaviors.

## 1. Introduction

Misperception of weight status, defined as under- or overestimating actual weight, is prevalent among adolescents in the United States (U.S.) [1,2]; approximately 30% of 8–15 year olds misperceive their weight status [2]. Characteristics associated with misperception of weight status in adolescents include their body mass index (BMI, kg/m^2^), gender, and race/ethnicity [2,3,4,5,6]. Although prevalence estimates vary by population, in general, adolescents with overweight or obesity tend to underestimate their weight, while those with normal weight may overestimate it [3,5,6]. Female adolescents are more likely than males to overestimate their weight status [4], while white adolescents are more likely to overestimate their weight status compared to their non-white counterparts [4,5]. Perception of weight status, independent of BMI status, is associated with poor weight management practices, including skipping breakfast and fasting, which may contribute to weight gain and the development of obesity [4,7,8,9,10,11,12]. It may also influence intakes of nutrients necessary for optimal growth and development [5,13,14,15,16].

Evidence from studies that investigate associations of adolescents’ weight status perception and diet (assessed as food groups, calories, and/or macro- and micronutrients) is inconsistent. In studies of adolescents ages 12 years and older, weight status perception was not related to intakes of low nutrient-dense foods (e.g., percent of calories from sweets and salty snacks), macronutrients (e.g., percent of calories from carbohydrates), or micronutrients (e.g., B vitamins, calcium, iron) [13,14]. However, among participants in the China National Health and Nutrition Survey (2004–2009), perception of being overweight was associated with higher calorie, fat, and protein intakes in 6–11 year olds but was not associated with intakes in 12–17 year olds [15]. Additionally, among a nationally representative sample of eighth grade students, those who underestimated their weight reported buying junk food and fast foods at school more frequently than those who accurately perceived their weights, although no statistically significant differences in reported consumption of soda, fruits, or vegetables were observed [5].

The possible influence of adolescents’ weight status perception on their nutrient intakes remains understudied in the literature, particularly among populations that are ethnically diverse and include adolescents of younger ages (prior to entering high school). In the current study, we examined associations of weight status perception, weight control intentions (e.g., desire to lose or gain weight), and nutrient intakes utilizing recently collected data (2005–2014) from a large, U.S. nationally representative sample of adolescents aged 10–15 years.

## 2. Methods

Data came from the National Health and Nutrition Examination Surveys (NHANES), 2005–2014. NHANES is a program of studies conducted by the National Center for Health Statistics designed to assess the health and nutrition status of non-institutionalized children and adults in the U.S. using a nationally representative sample. It consists of three components: an in-person interview, which includes collection of sociodemographic, dietary, and health-related information; a physical examination; and laboratory analyses [17]. Informed consent (oral assent and parental permission for children <12 years; informed assent for children ages 12–17 years) was obtained from all participants, and approval for the studies was obtained from the National Center for Health Statistics’ Research Ethics Review Board [18]. Information on NHANES and all procedures are extensively detailed elsewhere [19]. The current study was exempted from the human subjects review by the New York University Institutional Review Board.

Questions on weight status perception were added to the NHANES protocol beginning in 2005 and were only administered to children and adolescents ages 8–15 years. There were 7297 children and adolescents with information on weight status perception (2005–2014). Children who were 8–9 years old (n = 1869) were excluded from these analyses to focus on adolescents. Additionally, adolescents who were missing measured BMI (n = 32), underweight (BMI < 5th percentile, n = 171), missing dietary data (n = 251), or reported implausible energy intakes (e.g., likely not reflective of habitual intakes, <500 kcals/day, n = 18 or >5000 kcals/day, n = 16) were excluded. The final sample consisted of 4940 adolescents aged 10–15 years. Information on sociodemographic characteristics including gender, age, race/ethnicity (non-Hispanic white, non-Hispanic Black, Hispanic, or Other), and household income, defined by the poverty-to-income ratio (PIR) and categorized as low (<130%), medium (130≤350%), or high (≥50%), were collected during the in-person interview.

### 2.1. Measured and Perceived Weight Status and Weight Control Intentions

Height (nearest 0.001 meter, m) and weight (nearest 0.1 kilogram, kg) were measured by trained healthcare professionals as part of the NHANES physical examination and used to calculate BMI (kg/m^2^) and BMI percentiles [17,19]. Measured weight status was categorized as normal weight (5th≤85th BMI percentile), overweight (85th≤95th BMI percentile), or obese (≥95th BMI percentile) according to the Centers for Disease Control and Prevention growth charts [20]. Weight status perception was assessed using responses to the question ‘How do you consider your weight?’ (fat or overweight, too thin, or about the right weight). Weight control intentions were assessed using the responses to two questions: (1) ‘Are you trying to do anything about your weight’ (lose weight, gain weight, or stay the same weight/do nothing about weight) and (2) ‘In the past year, how often have you tried to lose weight?’ (never, sometimes, or a lot).

### 2.2. Dietary Intakes

Adolescents completed up to two 24-h dietary recalls. The first recall was collected in-person and the second recall was collected over the phone 3–10 days later. For adolescents aged 10–11 years, dietary recalls were completed with a parent/caregiver present in the room, while those aged 12 years and older completed the recalls alone. Approximately 90% of adolescents (n = 4466) completed both dietary recalls, and intakes from these recalls were averaged. Reported intakes of calories, percent of calories from protein, percent of calories from carbohydrate, percent of calories from fat, percent of calories from saturated fat, grams of sugar, and grams of fiber were considered in analyses.

### 2.3. Statistical Analysis

Stata version 15.1 (College Station, TX, USA) was used for all analyses. Bivariate associations between weight status perception and sociodemographic characteristics were tested using Pearson’s chi-square test or linear regression (for continuous variables). Multivariable linear regression was used to estimate associations of dietary intake variables with weight status perception and weight control intentions. All models were stratified by gender and adjusted for race, continuous age, BMI percentile, and PIR. Models for weight status perception were stratified by BMI status (dichotomized as normal weight and overweight/obese); though formal tests of statistical interaction were not significant (*p* > 0.05). Models for grams of sugar and fiber were also adjusted for total calories. All analyses were statistically weighted as required for the complex survey design of NHANES.

## 3. Results

The majority of adolescents were white, from households with a medium or high PIR, and normal weight; there were no differences in the distributions of these characteristics between boys and girls, as shown in Table 1. The mean (standard error, SE) for age and BMI percentile were 12.5 (0.05) years and 69.4 (0.01)% among boys and 12.5 (0.05) years and 70.5 (0.01)% among girls. Distributions of weight status perception and weight control intentions varied by measured weight status (BMI percentile categories: normal, overweight, or obese) and are provided in Table 2. The majority of boys and girls with normal weight or overweight perceived their weight as about right, while only 43% of boys and 31% of girls with obesity perceived their weight as about right. Weight control intentions, especially to lose weight, were prevalent (Table 2). Among boys with normal weight, overweight, or obesity, 25%, 65%, and 85%, respectively, reported trying to lose weight during the previous year, while among girls, 42%, 71%, and 92%, respectively, reported trying to lose weight in the previous year.

Associations of weight status perception and weight control intentions with dietary intakes among boys and girls are shown in Table 3 and Table 4, respectively. Among boys with overweight and obesity, those who perceived themselves as overweight reported consuming less sugar (−13.65 g, 95% CI: −23.06, −4.23) and more calories from protein (1.01%, 95% CI: 0.16, 1.87), compared to those who perceived themselves as just right. Among girls with normal weight, those who perceived themselves as overweight reported consuming less calories from saturated fat (−1.04%, 95% CI: −2.24, −0.17) compared to those who perceived themselves as just right.

Among boys, weight control intentions were associated with total calories, percent of calories from protein, and percent of calories from fat (Table 3). Compared to boys trying to stay the same weight, boys trying to gain weight consumed more calories (231.89, 95% CI: 102.51, 361.28). Boys who reported trying to lose weight a lot in the previous year consumed fewer calories (−188.34, 95% CI: −357.67, −19.01) and percent calories from fat (−1.41%, 95% CI: −2.80, −0.02) and greater percent calories from protein (1.48%, 95% CI, 0.41, 2.55) compared to those who never tried to lose weight. Weight control intentions were not associated with dietary intakes among girls (Table 4).

## 4. Discussion

Among a large, nationally representative sample of adolescents aged 10–15 years, perception of weight status as “about right” was highly prevalent among boys and girls, notably among those who were overweight or obese. Despite perceiving themselves as being at the right weight, many adolescents reported trying to lose or gain weight. Overall, weight status perception was not associated with dietary intakes in boys or girls, with the exceptions of lower consumption of sugar and percent calories from protein in boys with overweight or obesity who perceived themselves as overweight, and lower percent calories from saturated fat among girls with normal weight who perceived themselves as overweight. Weight control intentions were associated with dietary intakes in boys only. Compared to boys who never tried to lose weight, boys who reported trying to lose weight consumed fewer calories and percent of calories from fat and a greater percent of calories from protein.

In the current study, differences between boys and girls were observed for weight status perception and weight control intentions according to their measured weight status. Among boys with normal weight, 87% perceived themselves as just right, yet 25% reported trying to gain weight and 25% reported trying to lose weight in the previous year. Among girls with normal weight, 86% perceived themselves as just right, but 42% reported trying to lose weight in the previous year. This suggests that although adolescents may have a healthy weight, they are engaging in behaviors to change their weight status, especially weight loss. Similarly, among adolescents with overweight or obesity, the majority of girls and boys reported attempting to lose weight sometimes or a lot in the previous year. These observations are concerning, because adolescents, particularly within the age range of 10–15 years, are growing and undergoing other physical changes with high nutrient demands [16]. Given that unhealthy weight management strategies may be used by adolescents, it is important that adolescents are educated about healthy dietary and physical activity practices during this time [9,21,22].

In general, weight status perception was not associated with dietary intakes, except for overweight boys who perceived themselves as overweight, who consumed less sugar and more protein, and girls with normal weight who perceived themselves as overweight, who consumed less saturated fat. These findings are consistent with those from three other studies using nationally representative samples of adolescents. In a previous analysis of adolescents ages 12–18 years in NHANES III, weight status perception was not associated with intakes of energy, macronutrients, micronutrients, or low energy-dense foods, or serum concentrations of vitamins and carotenoids. The only exceptions were that boys who perceived themselves as overweight consumed less saturated fat (<10% of energy intake) and girls who perceived themselves as overweight consumed less calcium, compared to their respective counterparts who perceived themselves as being the right weight [13]. Among 9th–12th graders participating in the U.S. Youth Risk Behavior Surveillance Survey (YRBSS, 2001–2009) and among eighth graders, consumption of food groups, including fruits, vegetables, and soft drinks, did not differ by weight status perception [5,14]. However, perception of being overweight was associated with higher calorie, fat, and protein intakes among Chinese children and adolescents ages 6–11 years, although there were no associations observed for those ages 12–17 years [15]. Though all of these studies were cross-sectional, their results, taken collectively, suggest that weight status perception alone may not be strongly associated with dietary intakes.

Studies that examined associations of weight control intentions and dietary intakes in adolescents are limited. In the analysis of YRBSS participants, weight loss intentions (defined as trying to lose weight, through diet and/or exercise) were not associated with fruit/vegetable or soft drink intakes [14]. Similarly, among 8th graders, there were no differences in fruit, vegetable, soda, or fast food intake among those trying to lose weight compared to those not doing anything, but higher soda and fast food intakes were reported by those trying to gain weight. In the current study, intentions to change weight status (gain or lose weight) were associated with intakes of calories, fat, and protein among boys only. These findings contrast with those from a longitudinal analysis of the Project Eating Among Teens (EAT) study, which evaluated the relationship of healthful and unhealthful weight control behaviors with dietary intakes [23]. Among girls, persistent use of unhealthful weight control behaviors (over a 5-year period) was associated with less frequent meal consumption (e.g., breakfast), lower calorie intakes, and lower intakes of healthy dietary components, including fiber, calcium, iron, vegetables, and whole grains. In boys, persistent use of unhealthy weight control behaviors was only associated with less frequent meal consumption [23]. Discrepancies across studies are likely due to a range of factors, including study design, population characteristics, and data collection methods. The findings from the Project EAT study highlight the need for longitudinal studies of adolescents that follow them through the transitional period from late childhood/early adolescence to late adolescence/early adulthood.

Strengths of this study include the large sample size and nationally representative, ethnically-diverse population. The age range of 10–15 years allowed for focus on younger adolescents, which is understudied in the literature. Weight and height were measured by trained health professionals, which eliminates potential bias from self-reported values. Additionally, dietary intakes were represented as major nutrient intakes, which are reflective of food groups. For example, higher fiber intakes are generally associated with intakes of fruits, vegetables, and whole grains, while higher sugar intakes are generally associated with high energy-dense foods, such as confectionaries and sugar-sweetened beverages [24]. Analyses were limited by the cross-sectional design and method of dietary assessment; participants completed up to two 24-h recalls, which may not be reflective of usual diet. Weight perception and control intentions were based on three questions with limited response choices, which may not adequately discern them.

## 5. Conclusions

In this study, most 10–15 year olds perceived their weight as “about right” but still reported trying to change their weight, which was associated with some dietary intakes. These findings add to the current literature regarding how weight perception and weight control intentions may relate to dietary intakes in young adolescents. Future research should include longitudinal data collection encompassing a wider age range within adolescence, comprehensive dietary assessments, and detailed questions regarding weight control and eating practices.

## Figures and Tables

**Table 1:** Distributions of selected characteristics of adolescents ages 10–15 years, stratified by gender, participating in the National Health and Nutrition Examination Survey NHANES 2005–2014 (*n* = 4940).

Characteristic	Boys	Girls	*p*-Value ^a^
	*n* = 2493	*n* = 2447	
	%	%	
**Race/Ethnicity**			0.41
Non-Hispanic White	58	58	
Non-Hispanic Black	13	15	
Mexican	14	15	
Other	14	13	
**Household Poverty Income Ratio**			0.18
Low (<130%)	27	31	
Medium (130–349%)	36	35	
High (>=350%)	37	34	
**Measured WeightBMI Status**			
Normal Weight (5th–85th percentile)	59	59	0.99
Overweight (85th ≤ 95th percentile)	19	19	
Obese (≥95th percentile)	22	22	
	**Mean (SE)**	**Mean (SE)**	***p*-Value ^b^**
**Daily Dietary Intakes**			
Calories, Kilocalories	2191.61 (26.65)	1803.5 (18.3)	<0.001
Percent Calories from Carbohydrates, %	53.0 (0.27)	53.2 (0.27)	0.45
Percent Calories from Fat, %	33.0 (0.22)	33.3 (0.20)	0.16
Percent Calories from Saturated Fat, %	11.6 (0.08)	11.4 (0.09)	0.10
Percent Calories from Protein, %	15.1 (0.10)	14.6 (0.14)	0.004
Sugar, in grams	138.5 (2.7)	111.8 (1.5)	<0.001
Fiber, in grams	14.9 (0.24)	12.9 (0.16)	<0.001

NOTE: ^a^ *p* value from chi square test; ^b^ *p* value from linear regression.

**Table 2:** Distributions of weight status perception and weight control intentions, stratified by measured weight status (normal, overweight, or obese) and gender, among adolescents participating in the National Health and Nutrition Examination Survey 2005–2014 (*n* = 4940) ^a^.

Categories	Measured Weight Status			
	All	Normal	Overweight	Obese
	%	%	%	% ^b^
**Boys**				
**Weight Status Perception**				
About Right	76	87	80	43
Too Thin	7	12	0.4	1
Overweight	17	1	19	56
**Trying to Do Anything About Weight**				
Stay the Same Weight	55	66	54	24
Lose Weight	30	9	44	75
Gain Weight	15	25	2	1
**Tried to Lose Weight in Previous Year**				
Never	54	75	35	14
Sometimes	35	21	51	59
A Lot	10	3	14	26
**Girls**				
**Weight Status Perception**				
About Right	71	86	73	31
Too Thin	5	8	0.4	1
Overweight	23	6	27	68
**Trying to Do Anything About Weight**				
Stay the Same Weight	54	71	43	16
Lose Weight	41	21	57	84
Gain Weight	5	8	0.3	0
**Tried to Lose Weight in Previous Year**				
Never	41	57	28	8
Sometimes	47	37	59	64
A Lot	12	5	12	28

NOTE: ^a^ *p* values <0.05 for all comparisons across measured weight status category.

**Table 3:** Multivariable linear regression models estimating associations of weight status perception and weight control intentions with nutrient intakes ^a^ among boys ages 10–15 years participating in the National Health and Nutrition Examination Survey 2005–2014 (n = 2493).

		Calories		Percent Calories from Carbohydrate		Percent Calories from Protein		Percent Calories from Fat		Percent Calories from Saturated Fat		Sugar		Fiber	
	n	kcal	95% CI	%	95% CI	%	95% CI	%	95% CI	%	95% CI	grams	95% CI	grams	95% CI
**Weight Status Perception among Normal Weight**															
About Right	1201	**Ref**		**Ref**		**Ref**		**Ref**		**Ref**		**Ref**		**Ref**	
Too Thin	201	153.82	−37.28, 344.93	−0.99	−2.77, 0.79	−0.04	−0.75, 0.67	0.82	−0.73, 2.37	0.57	−0.18, 1.32	1.30	−10.72, 13.31	−0.98	−2.32, 0.37
Overweight	31	32.41	−318.13, 382.95	0.24	−3.15, 3.63	0.30	−1.11, 1.70	−0.45	−3.97, 3.08	0.19	−0.96, 1.33	−6.33	−23.07, 10.41	−0.13	−2.37, 2.10
**Weight Status Perception among Overweight/Obese**															
About Right	632	**Ref**		**Ref**		**Ref**		**Ref**		**Ref**		**Ref**		**Ref**	
Too Thin	11	−14.00	−480.29, 452.30	6.92	−2.68, 16.53	−0.83	−2.80, 1.15	−5.22	−14.48, 4.05	−0.93	−4.32, 2.47	10.81	−12.06, 33.68	0.16	−3.45, 3.77
Overweight	417	60.36	−83.00, 204.72	−1.63	−3.52, 0.26	1.01	0.16, 1.87 ^b^	0.39	−0.99, 1.78	0.35	−0.35, 1.04	−13.65	−23.06, −4.23 ^b^	−0.58	−1.60, 0.43
**Trying to Do Anything About Weight**															
Stay the Same	1237	**Ref**		**Ref**		**Ref**		**Ref**		**Ref**		**Ref**		**Ref**	
Lose Weight	855	−110.62	−228.55, 7.30	0.26	−0.95, 1.48	0.41	−0.26, 1.07	−0.68	−1.52, 0.16	−0.42	−0.52, 0.43	−2.47	−8.18, 3.25	−0.29	−1.10, 0.51
Gain Weight	399	231.89	102.51, 361.28 ^b^	0.68	−0.99, 2.34	−0.67	−1.33, −0.01 ^b^	0.02	−1.30, 1.33	0.17	−0.42, 0.76	8.25	−3.01, 19.51	−0.56	−1.57, 0.45
**Tried to Lose Weight in Previous Year**															
Never	1273	**Ref**		**Ref**		**Ref**		**Ref**		**Ref**		**Ref**		**Ref**	
Sometimes	937	−117.65	−226.07, −9.23 ^b^	−0.31	−1.37, 0.75	0.64	0.11, 1.18 ^b^	−0.29	−1.15, 0.57	−0.05	−0.45, 0.44	−0.31	−5.99, 5.36	0.32	−0.63, 1.28
A Lot	281	−188.34	−357.67, −19.01 ^b^	−0.03	−1.91, 1.84	1.48	0.41, 2.55 ^b^	−1.41	−2.80, −0.02 ^b^	−0.04	−0.73, 0.64	−2.65	−10.45, 5.15	−0.13	−1.48, 1.22

Ref, Reference; ^a^ All models are adjusted for body mass index percentile, race, age, poverty-to-income ratio, models for fiber and sugar are also adjusted for calories; ^b^ *p* < 0.05.

**Table 4:** Multivariable linear regression models estimating associations of weight status perception and weight control intentions with nutrient intakes ^a^ among girls ages 10–15 years participating in the National Health and Nutrition Examination Survey 2005–2014 (n = 2447).

Categories		Calories		Percent Calories from Carbohydrate		Percent Calories from Protein		Percent Calories from Fat		Percent Calories from Saturated Fat		Sugar		Fiber	
	n	kcal	95% CI	%	95% CI	%	95% CI	%	95% CI	%	95% CI	grams	95% CI	grams	95% CI
**Weight Status Perception among Normal Weight**															
About Right	1187	**Ref**		**Ref**		**Ref**		**Ref**		**Ref**		**Ref**		**Ref**	
Too Thin	117	2.02	−188.99, 193.04	−0.44	−3.04, 2.16	0.20	−0.81, 1.21	0.10	−2.07, 2.27	0.20	−0.72, 1.12	−5.79	−16.04, 4.46	0.16	−1.06, 1.38
Overweight	71	−39.93	−377.28, 297.42	0.22	−2.70, 3.14	0.95	−1.04, 2.94	−1.50	−3.31, 0.31	−1.04	−2.24,−0.17 ^b^	−2.49	−14.20, 9.22	−0.29	−1.92, 1.34
**Weight Status Perception among Overweight/Obese ^c^**															
About Right	524	**Ref**		**Ref**		**Ref**		**Ref**		**Ref**		**Ref**		**Ref**	
Overweight	542	−90.92	−242.58, 63.74	−0.45	−2.54, 1.64	−0.05	−0.96, 0.86	0.26	−1.33, 1.85	−0.46	−1.14, 0.23	−0.71	−8.20, 6.78	0.42	−0.65, 1.49
**Trying to Do Anything About Weight**															
Stay the Same	1235	**Ref**		**Ref**		**Ref**		**Ref**		**Ref**		**Ref**		**Ref**	
Lose Weight	1079	−56.86	−157.24, 43.51	−0.47	−1.77, 0.83	−0.06	−0.60, 0.48	0.37	−0.64, 1.38	−0.24	−0.70, 0.23	−2.41	−7.81, 2.99	−0.13	−0.77, 0.51
Gain Weight	133	9.25	−166.06, 184.56	0.05	−2.78, 2.88	−0.33	−1.14, 0.49	0.23	−2.26, 2.72	0.04	−0.91, 0.99	−3.80	−16.13, 8.54	−0.50	−1.65, 0.65
**Tried to Lose Weight in Previous Year**															
Never	1023	**Ref**		**Ref**		**Ref**		**Ref**		**Ref**		**Ref**		**Ref**	
Sometimes	1146	−5.93	−107.06, 95.20	0.02	−1.40, 1.44	−0.48	−1.06, 0.09	0.51	−0.52, 1.54	0.06	−0.39, 0.51	0.30	−4.86, 5.47	0.10	−0.53, 0.72
A Lot	277	−18.21	−132.72, 96.29	0.64	−1.37, 2.64	0.08	−1.03, 1.20	−0.70	−2.28, 0.87	−0.49	−1.21, 0.23	1.12	−6.41, 8.65	0.25	−0.55, 1.05

Ref, Reference; ^a^ All models are adjusted for body mass index percentile, race, age, poverty-to-income ratio, models for fiber and sugar are also adjusted for calories; ^b^ *p* < 0.05; ^c^ There were only six girls who were overweight or obese and reported that they were too thin.

## Abbreviations

NHANES	National Health and Nutrition Examination Surveys
BMI	body mass index
